# High resolution skin-like sensor capable of sensing and visualizing various sensations and three dimensional shape

**DOI:** 10.1038/srep12997

**Published:** 2015-08-13

**Authors:** Tianbai Xu, Wenbo Wang, Xiaolei Bian, Xiaoxue Wang, Xiaozhi Wang, J.K. Luo, Shurong Dong

**Affiliations:** 1 Dept. of Info. & Electron. Eng., Zhejiang University, 38 Zheda Road, Hangzhou, China; 2 Inst. of Renew. Energ. & Environ. Tech., University of Bolton, Deane Road, Bolton, U.K

## Abstract

Human skin contains multiple receptors, and is able to sense various stimuli such as temperature, pressure, force, corrosion *etc*, and to feel pains and the shape of objects. The development of skin-like sensors capable of sensing these stimuli is of great importance for various applications such as robots, touch detection, temperature monitoring, strain gauges etc. Great efforts have been made to develop high performance skin-like sensors, but they are far from perfect and much inferior to human skin as most of them can only sense one stimulus with focus on pressure (strain) or temperature, and are unable to visualize sensations and shape of objects. Here we report a skin-like sensor which imitates real skin with multiple receptors, and a new concept of pain sensation. The sensor with very high resolution not only has multiple sensations for touch, pressure, temperature, but also is able to sense various pains and reproduce the three dimensional shape of an object in contact.

Human skin is a complex and sensitive system containing multiple receptors. These receptors act as sensors, and are able to sense various stimuli such as thermal variation, light touch, mechanical pressure, deformation and chemical corrosion, with very high sensitivity and resolution^1^. The development of skin-like sensors or electronic-skin (e-skin) with the capability of monitoring and sensing stimuli has attracted great attention for various applications, including robots^2^,^3^, touch detection^4^,^5^,^6^, temperature monitoring^7^, strain gauges^8^,^9^,^10^, medicine and healthcare^11^,^12^. The sensors based on the mechanisms of piezoresistivity, capacitance, resistance and switch arrays have been developed^13^,^14^,^15^,^16^, demonstrated their great potential for applications. Among various mechanisms, the piezoresistive and resistive mechanisms are preferred as the changes induced by many stimuli such as strain, pressure, force and temperature can be converted to a change in resistance which can be simply detected by peripheral circuitry. Sensors based on piezoresistive and resistive mechanisms can also be made with small dimensions and low signal noise, thus they can deliver a high resolution for sensation with high sensitivity. Bao *et al.* reported a highly sensitive touch sensor using microstructures^17^. Webb *et al.* showed a thermally sensitive skin-like sensor with precision thermal characteristics. A user-interactive electronic skin has been demonstrated which is able to visualize pressure distribution using an array of organic light emission diodes^18^. Other materials with featured functional characteristics have also be investigated, *e.g.* thermo-responsive polymers^19^, self-healing materials^20^,^21^ and so on. However the skin-like sensors so far developed are far from perfect as the majority of them focus on only sensing one stimulus such as pressure, strain or temperature. Besides the functions mentioned above, however, human skin can also sense various pains^22^ that are an extreme case of any somatosensory experience imposed on the skin by one of stimuli^23^, and feel and reproduce the shape of objects in contact. Unfortunately, the skin-like sensors so far developed are not trying to imitate various pains and reproduce the shape of object in contact, these make the current sensors significantly inferior to the human skin. Here we report a high resolution and high performance skin-like sensor which aims to imitate all the functions of a human skin, not only detect multiple-sensations induced by temperature, touch and pressure simultaneously with very high sensitivity, but also visualize the distribution of the sensations. This paper, for the first time, focuses on resolving high resolution deformation patterns of a skin-like device and mapping them for different stimuli. It can also reproduce the three dimensional (3D) shape of an object in contact. This sensor replicates most of the receptors existing in human skin, and thus it paves the way for the development of intelligent skin for practical applications.

## Results

### Device fabrication and sensation visualization

**Figure 1:**
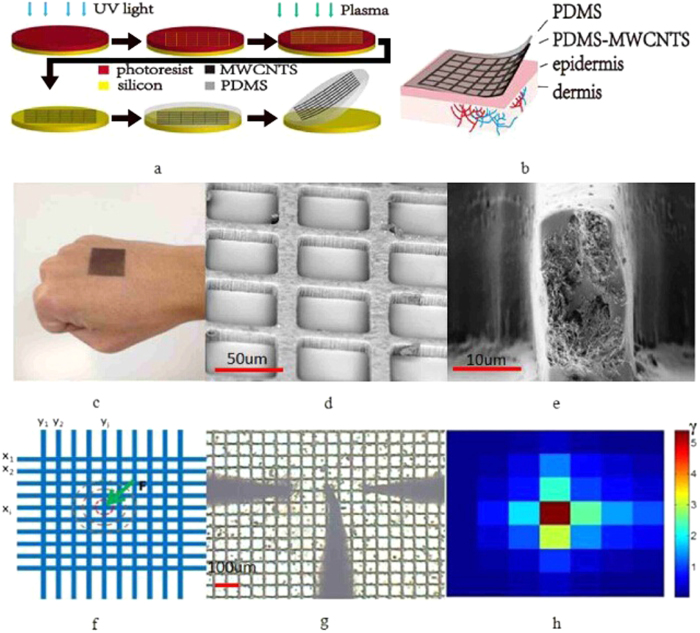
(**a**) Fabrication process for the E-skin. (**b**) A schematic diagram of the skin-like sensor. (**c**) a photo of the fabricated senor on a hand. (**d,e**) SEM images of the PDMS/MWCNTs networks and the cross section of a conductive line. (**f**) A measurement scheme used to map the variation of resistance. (**g**) A photo of the sensor under test using three probes. The mid probe was used to press the conductive line, while the other two were used to measure the resistance change, and (**h**) is the reproduced color mapping of resistance change, hence the deformation induced by the probe tip. The color of the unit is shown in the scale bar on the right of the color map, red color present the larger change of the resistance, the resistance change in the blue area is smaller.

The skin-like sensor consists of a crisscross conductive network as the receptors (sensors) and a polydimethylsiloxane (PDMS) surface layer as the dermis. The conductive network was made from a multiwall carbon nanotubes (MWCNTs) and PDMS composite. The processing flow for device fabrication is shown in Fig. 1a, and the details are described in the Methods section. A comparison of the stiffness of the human skin and the skin-like sensor is shown in the supplementary information (SI).

The materials and device structures were characterized by scanning electron microscope (SEM) (S4800, Hitachi). In order to test the performance of the sensor, the sensor was connected to the peripheral circuitry. The circuit design is presented in the SI. The skin-like sensor was cut into 4 × 4 cm^2^ square. Each side of the sensor has 30 electrodes for electrical connection. For the convenience of sensing, the resistance change of 20 pairs of the 30 pair electrodes was measured in the middle part of the sensor, and the sensor was attached to the electrodes of a peripheral circuitry using the conductive silver gel.

Figure 1b,c show the microphotos of the sensor. Figure 1d,e are the SEM pictures, showing the details of the conductive crisscross network and the cross section of a conductive line. MWCNTs were used to form the conductive network, and the change of resistivity of the network was used to sense various stimuli. The skin-like sensor structure is robust, flexible and biocompatible. The more detailed process flow can be found in Methods.

The resistance of conductive lines of the as-made sensor is in the range of 18 kΩ to 21 kΩ, mostly caused by the variation of the MWCNTs content in the channels. The resistances of the sensor vary with cyclic extension due to the relaxation of the composite and the settling down of the CNTs; therefore stabilization was applied before sensing experiments to ensure the change of the resistivity is caused by a stimulus rather than by the relaxation. The resistance of the conductive lines increases slowly with the cyclic extension and eventually stabilizes (Figure S2). Once settled down, the resistance of the conductive lines was found to increase almost linearly with the increase of strain in a range less than 40%, and gradually saturates with further extension. The pressure and touch sensing experiments were conducted mostly in the linear region, while the thermal and pain sensation tests are not limited by the linear relationship.

When the sensor is deformed or damaged by a stimulus such as touch or corrosion, it not only changes the resistance of the conductive line, but also affects the resistance of the surrounding lines, generating a two dimensional (2D) distribution of resistance variation. Assume the resistance of the *ith* conductive line is Rx_i_ in the x-direction as indicated in Fig. 1f, and Ry_i_ in the y-direction, the resistance change before and after the application of a stimulus is


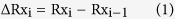


and


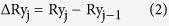


respectively. The ratio


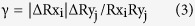


will represent the change of the resistance at the point of the *ith* and *jth* lines perpendicular to each other with the intensity representing the strength of the stimulus. This ratio will be used to visualize various sensations in 2D in this work. An absolute value of |ΔRx_i_| was used to assess the change ratio, as it can be used to distinguish the type of stimuli as discussed later.

Figure 1g,h show a photo of a 7 × 7 conductive line network (note a small area of the sensor and probes were used to clearly demonstrate the distribution and measurement of the resistance) used for the resistance measurements and the mapping of the resistance change ratio, γ. The middle probe was used to induce the deformation, while the left and right probes were used to measure the resistance of the lines. The line pressed by the probe has the largest deformation, hence the largest change of the resistance; and the change of the resistance decreases gradually as the lines move away the deformed point, producing a 2D mapping of the resistance change. This 2D mapping also shows the high resolution of the sensor developed, and the resolution can be improved by scaling down the space of the lines or by utilizing other structures^24^. The dynamic response of the skin-like sensor to a force applied is very fast, in the order of milliseconds, limited by the measurement system. An example of dynamic response of one conductive line is shown in Figure S3, showing the fast response of the sensor. Generally, humidity would affect the conductivity of the CNTs films, hence the sensitivity of the sensor. However the CNTs films were sealed by a “thick” PDMS cover, preventing the moisture entering into the CNTs films, thus eliminating the humidity effect on our sensors. Also absorption of moisture by PDMS was found not changing the CNTs film conductivity.

### Imitation of touch and pressure sensations

**Figure 2:**
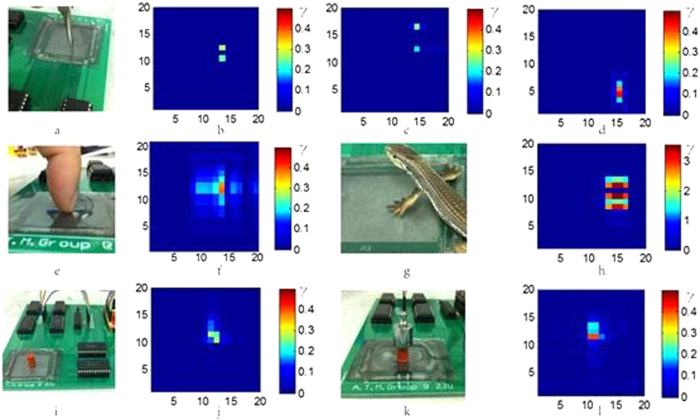
(**a–d**) Two-point threshold touch experiments with a distance of 1, 2 and 4 mm, respectively. The intensity of the color increases as the distance is reduced because of the combined effect from the two points increases. (**e,f**) Pressure sensing experiment by a finger, and (**g,h**) by a model lizard. Both show the rough shape of the touching objects. (**i–l**) comparison of pressures induced by an identical object but with different weight of 0.25 g and 10.25 g. The resultant color patterns are similar, but the intensity increases significantly when a heavier object was placed on the sensor.

A two-point threshold method is normally used for touch sensation which may vary for different parts of a human body. The smallest distance is typically 1 ~ 2 mm, existing on the lip and finger tips^25^. Figure 2a shows the photo of a two-point touching experiment, and Fig. 2b–d show the color mappings of the touch sensation imposed by two metal pins with a spacing of 1, 2 and 4 mm, respectively. The bright color represents the touch points, and these fade into the background of the dark blue. For the two points with a spacing of 1 mm, the two bright spots are next to each other with higher intensity. As the spacing between the two pins increases, the corresponding color intensity decreases. Thus touching of the two points at different distances has been successfully detected and visualized.

A large force over a relatively large area is needed to activate the pressure receptor which lies deep in the skin. Figure 2e,f are a photo of a finger tip pressed on the sensor and the corresponding color mapping. The mapping clearly shows the areas deformed by the finger pad and the nail. Figure 2h shows the mapping imposed by the feet of a model lizard (Fig. 2g) with a rough shape of the paw of the lizard. The pressure sensitivity of a skin-like sensor is very important for various applications. To test this, two objects with the same shape but different weights of 0.25 and 10.25 g, respectively, were placed on the sensor (4 × 4 cm^2^) for sensing as shown in Fig. 2I,k. The heavier object produces a more obvious change in resistance than that by the light object. The touch area is 0.44 cm^2^. The pressure for the small object is 55.7 Pa, corresponding to a pressure sensitivity (defined as ΔR/RΔP) of 3.6 kPa^−1^. This detection limit is much smaller than the smallest pressure (>100 Pa) human skin can sense, though larger than some values reported by others^26^, clearly demonstrated the extraordinary sensitivity of the sensor developed. A pressure may squeeze the PDMS/MWCNTs networks, making better conductive and leading to the decrease of the resistance. But the pressure may also elongate the conductive line, leading to the increase of the resistance. The elongation of the conductive lines is believed to be the dominant effect for our skin-like sensor.

### Imitation of thermal sensation

**Figure 3:**
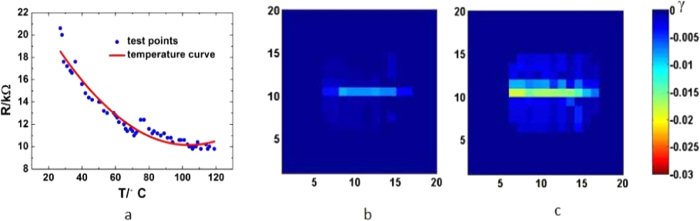
(**a**) Resistance variation as a function of temperature for one conductive line of the skin-like sensor. It decreases with the increase in temperature. (**b,c**) The mapping of the resistance variation of the sensor induced by a heated wire of 35 °C and 45 °C, respectively. The areas with the hot wire on it have been reproduced and the intensity is higher for the hotter wire. Note very small resistance change was sensed by the skin-like sensor, showing the high thermal sensitivity of the device.

Thermal sensation of skin is a sense that is associated with our need to maintain our internal temperature, and to warn potential harm when the surrounding temperature is too high. The skin-like sensor is able to sense temperature variation owing to the resistivity change of the conductivity lines with temperature. A typical change of the resistance from one conductive line is shown in Fig. 3a which is a nonlinear relationship and varies from sample to sample slightly, depending on the CNT content and process conditions. The temperature coefficient of resistance (TCR, i.e. the temperature sensitivity which is defined as TCR = ΔR/RΔT) is in the range of −12000−ppm/°C to 4000 ppm/°C. The resistance of the MWCNT/PDMS composite decreases monotonically as the temperature is increased which has been explained by a heterogeneous fibrillar mode^27^,^28^, opposite to what is observed by the application of a pressure. This provides us with a means to distinguish the thermal effect from the sensations induced by other stimuli.

To imitate the sensation of temperature, a heated thick wire of 1 mm in diameter was put on the sensor on a flat surface. The color mappings of the resistance change for the sensor induced by temperatures of 35 and 45 °C are shown in Fig. 3b,c. The resistances decrease as the temperature is increased, in agreement with the observation shown in Fig. 3a. As the resistance change can be detected by the circuitry in a very short time, the shape of the heated wire can be demonstrated clearly. The bright color represents the area where the resistance decreases, and the dark blue areas are not affected by the heated wire. The shapes of the color mappings are consistent with the shape of the wire, and an increase of temperature by 12 °C (the room temperature was 23 °C) has been successfully detected by the sensor. The intensity of the change increases with temperature, clearly demonstrating its high sensitivity to thermal stimulus.

### Imitation of pain sensations

**Figure 4:**
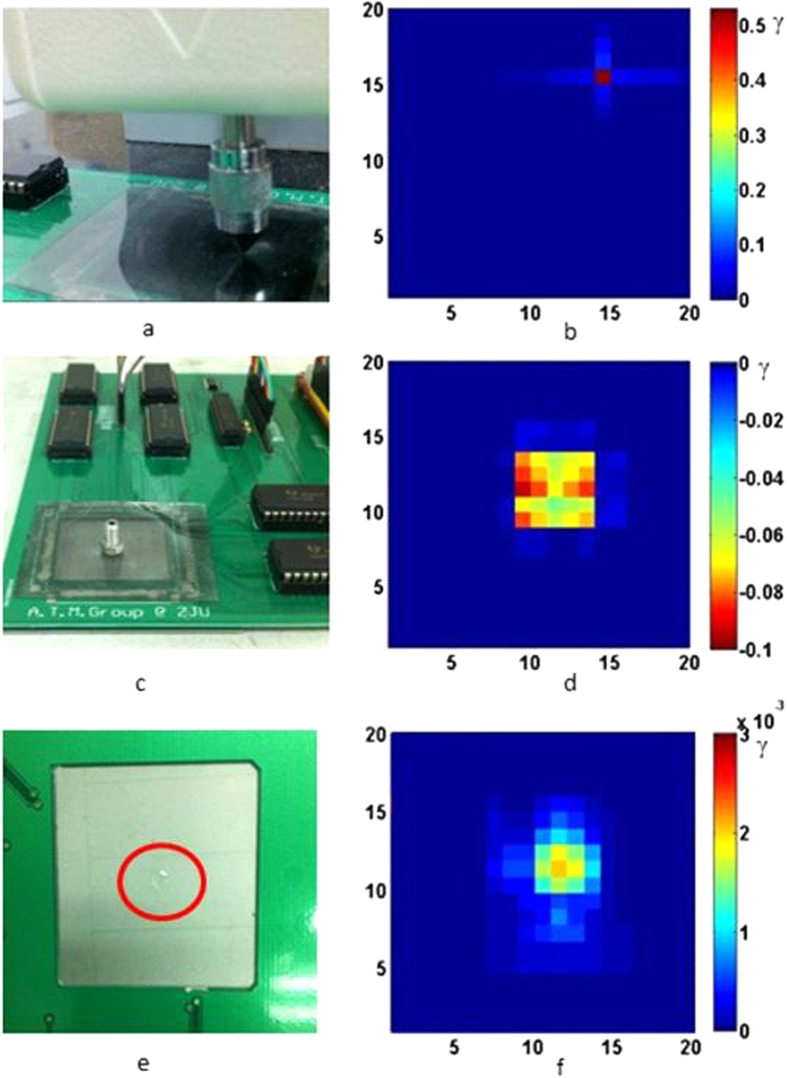
(**a**) a photo of mechanical pain imitation and (**b**) the color mapping. (**c**) A photo of a heated metal bolt used for thermal pain imitation and (**d**) the color mapping. (**e**) A photo of a chemically corroded skin-like sensor used for chemical pain imitation and (**f**) the color mapping of the resistance change.

Pain is an extreme sensory experience felt by skin. When exceeding a threshold, people feel pain as an alarm of possible damage to body^29^. Mechanical, chemical and thermal stimuli all can induce a pain sensation^30^. Here we introduce a new concept of pain sensation for the skin-like sensor in this work and try to detect three kinds of extremes of stimuli which imitate pain sensations by the sensor. For the imitation of pain induced by a mechanical stimulus, a young male volunteered for the experiment to determine the pain threshold. The experiment was conducted on the back of his hand using a force gauge with a metal tip with the size of 0.6 mm^2^. The average force that brought a pain sensation was about 1.98 N. Therefore a force of 2.0 N (corresponding to a pressure of 3.33 × 10^6 ^Pa) from the force gauge was then applied to the sensor on a flat surface (Fig. 4a), and the color mapping result is shown in Fig. 4b. The deformation area is a single point, and the change of the resistance is visualized by a single bright point, replicating the pain sensation by the skin-like sensor. In principle, this type of pain and touch or pressure are all caused by mechanical deformation, but the deformation related to the pain is much larger and sharper, or may be an extreme distortion of the surface. The deformation caused by touch or pressure is much smoother and the contrast in the color mapping is not as sharp as that of the pain sensation. Clear definition for both types of sensation is difficult, and is also dependent on the individual and sensitivity of various body parts.

People feel pain when the temperature of an object touching the skin is above 50 °C. From Fig. 3c, we can roughly define a threshold of −0.03 for thermal pain. The absolute value of this is smaller than that of mechanical pain, but its sign is opposite, providing us with a means to judge whether it is a mechanical pain or thermal one. A metal bolt heated at 100 °C was placed on the sensor (Fig. 4c), and the color mapping of the resistance change is shown in Fig. 4d. The resistance of the heated area decreases as expected, and the intensity (minus value) is much larger than that induced by a 45 °C wire shown in Fig. 3c, clearly demonstrated the thermal pain sensation sensed by the sensor. It is interesting to note that the intensity in the middle area of the colored domain is lighter than those of the surrounding. It is attributed to the combined effect of pressure and temperature. A high temperature decreases the resistance, while that induced by pressure (the bolt weight) gives the opposite effect. As the pressure in the middle area is much larger than the surrounding area due to the curvature of the surface of the bolt, the pressure induced resistance increase offsets some of the resistance decrease induced by the raised temperature. As a result, the intensity in the middle is lower than the surrounding.

Chemical corrosion to skin will cause a sensation of pain and can be detected by chemoreceptors in the skin. Chemicals will corrode and damage the PDMS surface and distort the conductive network, and can be utilized to develop chemoreceptor-based sensors for the skin-like sensor. To demonstrate this, sulfuric acid (49%) was used to imitate the pain induced by the corrosion and distortion of the epidermis as shown by the photo of Fig. 4e. The damage and break-off of the conductive network result in a change of the resistance in 2D as shown in Fig. 4f. The resistance of the damaged area increases, and the visualized shape is consistent with the damaged area, clearly reproduced the pain sensation by the sensor. Sulfuric acid can etch PDMS but not effectively, whereas CNTs are chemically inert. Therefore the resistance change is not caused by the corrosion of the MWCNTs/PDMS conductivity of the lines, but the distortion of the conductive path of the lines when the PDMS surface layer was removed. To increase the sensitivity for the chemoreceptor, relatively chemical-sensitive polymers or conductive nanoparticles can be used. This will be investigated in future. It has to be pointed out that the chemical corrosion induced pain sensation is very preliminary, and it is also destructive and not recoverable, thus it is not particularly suitable for practical application. But this work provides a new concept and has demonstrated the possibility of chemoreceptors in the skin. More work is needed to develop skin-like sensor with better chemoreceptors.

### Sensing and reproducing object shape

**Figure 5:**
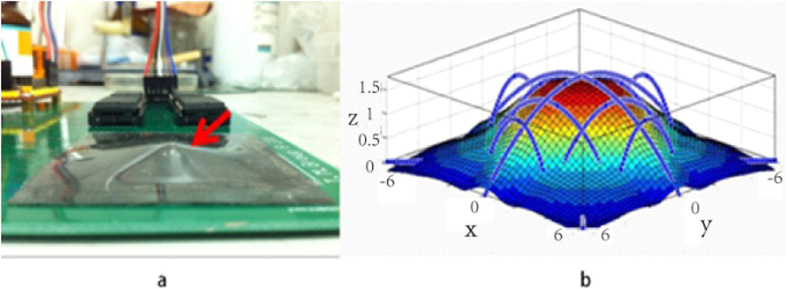
(**a**) A piece of skin-like sensor was placed on top of a ball with the diameter of 5 mm and deformed under a force. (**b**) The reproduced shape of the top part of the ball in contact through sensing and mathematic treatment, clearly demonstrated the ability to reproduce the shape.

An ideal skin-like sensor should, not just sense stimuli, but also “feel” and “reproduce” the shape of an object in touch. Here we demonstrate this feasibility by our E-skin using a ball as a demonstrator as shown in Fig. 5a. The deformation was limited to 5 mm in depth for a piece of 4 × 4 cm^2^ skin-like sensor. The elongation length of each sensing line can be obtained through the measurement of the resistance change. For simplicity, three conductive lines were chosen from the nine lines for the measurements that cover the center and the edges of the deformed area as shown in Fig. 5b. Based on the measured results, a model was then established to reproduce the surface profile of the object. The deformation generated by the ball is limited to the top part of the ball and therefore can be represented by a quadratic function approximation as follows,


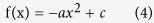


here a and c are constant. With this relationship, we can obtain sufficient points from the surface of the ball to reproduce the shape. The values of the constant a and c can be calculated by solving the equations.


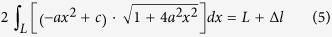



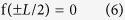


where ΔL is the increment of the length, and L is the initial length. L and ΔL are known since the length of the quadratic curves can be estimated from the resistance change measured. The deformation detected by three lines in the x-direction would be the same as those in the y-direction owing to the symmetric structure, therefore the calculation can be further simplified with only three conductive lines measured. Figure 5b shows the reproduced surface profile of the ball, similar to the upper part of the ball. For objects with a vague shape, reproduction of the surface profile by a model would be extremely difficult. However measurements of a sufficient number of points of the shape change, especially the key locations, would make the reproduction of the shape of an object possible. This will be investigated in future.

## Discussion

The results have demonstrated that the skin-like sensor is capable of detecting and distinguishing sensations induced by different stimuli, and sensing the brief 3D shape of a ball. This paves the way for practical applications. However identification of the types of sensations, especially the pain sensations, requires some judgment. Both the mechanical and corrosion pains are correlated to the positive change of the resistance, while the thermal pain is associated with the negative change of the resistance, allowing the identification of thermal pain. Mechanical pain is associated with the deformation which is recoverable once the force is removed, while corrosion pain is associated with permanent change of the resistance. Therefore, the pain sensations can be distinguished and can be utilized for the development of intelligent skin-like sensor with self-justification for sensations. However, when there are both mechanical and thermal inputs, it is difficult to differentiate the type of stimulation sources. The problem may be solved by integrating more device structures. For example, fractal design^31^ could be introduced to eliminate the effect of the mechanical input, meanwhile the sensor with such a structure can also respond to the thermal input as well. However more research is needed to distinguish different inputs simultaneously. In conclusion, we have developed a skin-like sensor technology which imitates real skin with multiple receptors with high resolution. We have also introduced a new concept of pain sensation by this sensor. The skin-like sensor has very high resolution, and not only has various sensations for touch, pressure, temperature, but also is able to sense various pains and to reproduce the three dimensional shape of an object in contact.

## Methods

### Device fabrication

The MWCNTs were purchased from Chengdu Organic Chemical Co. Ltd, China. They have the length distribution of 10 μm to 20 μm, the inner diameter of 5 nm to 15 nm and the outer diameter of 50 nm to 80 nm. The PDMS (184 Silicone Elastomer, Dow corning Co. Ltd) based flexible skin-like sensor were fabricated by the following process steps: (1). Crisscross network channels were patterned by photolithography process using positive photoresist (AR-P 5350) on a 4-inch silicon substrate with the size of 4 × 4 cm^2^. It was then etched by deep reactive ion etch (DRIE, Plasmalab System 100, Oxford Instrument) to form the crisscross network channels of 50 μm deep and 10 μm wide. The Si wafer was used as the mould to fabricate the sensor. (2). The network channels of the silicon mould were filled with MWCNTs powder with shake, and then the excessive CNTs above the surface of the Si wafer were removed using a razor. The weight of the MWCNTs was ca. 8.5 mg (±10%) after the excessive nanotubes were removed. (3). The silicon mould with MWCNTs in the channels was filled with the degassed prepolymer PDMS (at a ratio of 10:1 for the base to the crosslinker by mass) with the amount prefixed for a sensor and waited for 10 min to let the PDMS penetrate into the gaps of the MWCNTs in the channels. It was then spun at 1000 rpm for 3 sec to obtain a layer of PDMS with a fixed thickness on top of the composite in the channel. (4). The PDMS composite on the Si mold was then solidified in an oven at 90 °C for 30 min. (5). Finally the PDMS E-skin was peeled off the silicon wafer, ready for use.

The cross section of the PDMS/MWCNTs composite lines is 50 × 10 μm^2^, while the thickness of the top PDMS support layer is 250 μm. MWCNTs were used to form a conductive network, and the change of resistivity of the network was used to sense various stimuli.

### Characterization

Device structures and the details of the conductive lines were characterized by scanning electron microscope (SEM) (S4800, Hitachi). The resistance measurements were conducted using a circuitry specially designed for this work or by the probe station (Vector MX100 DC) and the Agilent B1500A semiconductor device analyzer. For temperature sensation experiments, a hot plate (C-MAG HP 4, IKA) was used which is able to control the temperature down to 1 °C.

The samples were cleft into 4 × 4 cm^2^ pieces for the sensation experiments. The peripheral circuitry was composed of a microchip (MSP430F149), an A/D converter (ADC7824), 4 decoders (CD4514B) and 4 multiplexers (CD4067B). The large pieces were cramped at the two ends by an electromechanical testing machine (HSV, Handpi Instruments Co. Ltd). The force gauge (HP, Handpi Instruments Co. Ltd) with the precision of 0.001N was used to apply forces on the E-skin in the pain sensation imitation.

## Additional Information

**How to cite this article**: Xu, T. *et al.* High resolution skin-like sensor capable of sensing and visualizing various sensations and three dimensional shape. *Sci. Rep.* **5**, 12997; doi: 10.1038/srep12997 (2015).

## Supplementary Material

Supplementary Information
