# Supplementary material for: High resolution skin-like sensor capable of sensing and visualizing various sensations and three dimensional shape
Source: Sci Rep. 2015 Aug 13;5:12997. doi: 10.1038/srep12997 (PMC4534778; doi:10.1038/srep12997)
Supplement: Supplementary Information [file srep12997-s1.doc]

Supplement Information

**High resolution skin-like sensor capable of sensing and visualizing various sensations and three dimensional shape**

Tianbai Xu^1^, Wenbo Wang^1^, Xiaolei Bian^1^, Xiaoxue Wang^1^,Xiaozhi Wang^1^*, J.K.Luo^2,1^ and Shurong Dong^1^

Dept. of Info. & Electron. Eng., Zhejiang University, 38 Zheda Road, Hangzhou, China

Inst. of Renew. Energ. & Environ. Tech., University of Bolton, Deane Road, Bolton, U.K.

1. **Comparison of the stiffness of the human skin and the skin-like sensor**

The mechanical stiffness of the skin-like sensor depends on the stiffness of the PDMS, as the thickness of the PDMS substrate was 40 times thicker than that of the PDMS-CNTs sensing network. According to the experiment result, the skin stiffness is ranging from 0.64 N/mm to 3.74 N/mm, which varies from different part of the body[^1^](#_ENREF_1). The stiffness of the PDMS (at a ratio of 10:1 for the base to the crosslinker by mass) is 3N/mm, which was in the range of the stiffness of the human skin[^2^](#_ENREF_2). And the stiffness of the sensor can be tuned by changing the ratio of the base to the crosslinker of PDMS. More base and less crosslinker lead to the lower stiffness. Other ways to represent the mechanical stiffness of the skin-like sensor were elastic modulus and the shore A hardness. The comparison result of the skin and the sensor was shown in the tableS1.

**TableS1 The comparison of the mechanical stiffness of the skin-like sensor and skin**

|  | elastic modulus(MPa) | Stiffness(shoreA hardness) | stiffness (N/mm) |
| --- | --- | --- | --- |
| PDMS(10:1,base to crosslinker) | 2.6 | 43 | 3 |
| skin | 2.1[^3^](#_ENREF_3) | 20~40[^4^](#_ENREF_4) | 0.64~3.74 |

1. **The repeatability of the fabrication process**

In order to test the repeatability of the fabrication process and devices, one pair of electrodes separated by 2 cm were randomly chosen from each device of nine samples to measure the resistance and the result is shown in Figure S1. The resistances of individual conductive lines of the as-made skin-like sensor are typically between 18 kΩ and 24 kΩ, and vary from line to line, and the largest RSD is less than 10% which determines the accuracy of the measurements and sensation experiments. The variation of the resistance of the conductive lines is relatively small as compared to the absolute value of the resistance, and is mainly caused by the variation in the fabrication process, which could be improved by further narrowing the distributions of the size and electronic property of MWCNTs.


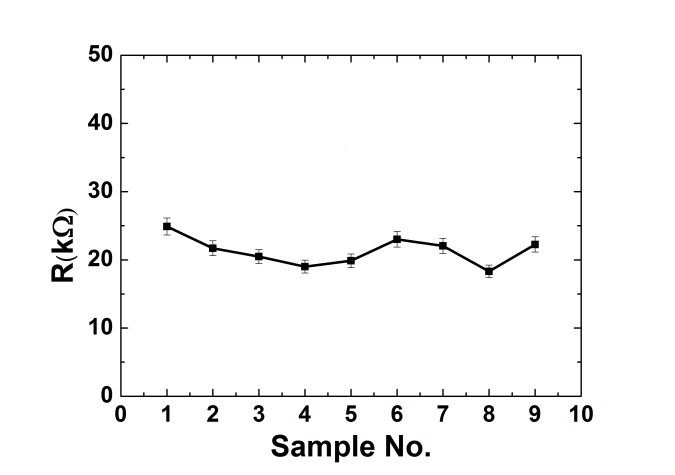


**Figure S1.**The resistance of the fabricated conductive lines varies from sample to sample, but the variation is rather small compared the absolute value of the resistances, demonstrated its repeatability of the skin-like sensor fabricated.

1. **Stretchability and stability**

To see the stretchability of the skin-like sensor, the resistance of the skin-like sensor was tested under an extension up to 200% for up to 5000 times. No physical damage to the network was observed. However the extension was found to increase the resistance of the conductive lines due to the relaxation or re-arrangement of CNTs in the composite and polymer relaxation etc. Figure S2a shows the resistance changes of nine conductive lines of a piece of the skin-like sensor extended by 100% for 5000 times. The resistances increase with the cyclical stretch slowly for all the lines though the degree varies from one line to another. The resistance of each conductive line is mostly between 20 kΩ and 25 kΩ after 5000 times stretch with the largest deviation of about 6.2%. The deviation is believed to be associated with the initial value of the resistances of the lines, and can be reduced by process improvement. The maximum values, minimum values and the mean values recorded after every 1000 times extension are summarized in Table S2 for clarity.

**Table S2.****Summary of resistance of a piece of skin-like sensor after 5000 times stretch.**

|  | 1 | 2 | 3 | 4 | 5 | 6 | 7 | 8 | 9 |
| --- | --- | --- | --- | --- | --- | --- | --- | --- | --- |
| Max (kΩ) | 21.50 | 23.20 | 23.70 | 25.10 | 23.60 | 24.80 | 25.1 | 21.7 | 23.7 |
| Min (kΩ) | 19.30 | 19.80 | 20.00 | 21.00 | 20.90 | 21.40 | 23.8 | 19.9 | 20 |
| Mean (kΩ) | 20.06 | 20.90 | 21.34 | 22.39 | 22.03 | 22.40 | 24.1 | 20.75 | 22.17 |
| Variance | 0.55 | 1.34 | 1.56 | 1.94 | 1.03 | 1.18 | 1.77 | 0.61 | 1.69 |
| Std Deviation | 0.74 | 1.16 | 1.25 | 1.39 | 1.02 | 1.09 | 1.33 | 0.78 | 1.30 |
| RSD | 3.70% | 5.53% | 5.86% | 6.22% | 4.61% | 4.85% | 5.52% | 3.76% | 5.86% |

Figure S2b is the variation of resistances of 9 lines after the stabilization process (extended by 100% for 1000 times) as a function of extension. The resistances of the conductive lines increase almost linearly with the strain up to 40%, and then gradually increase slowly with further extension.


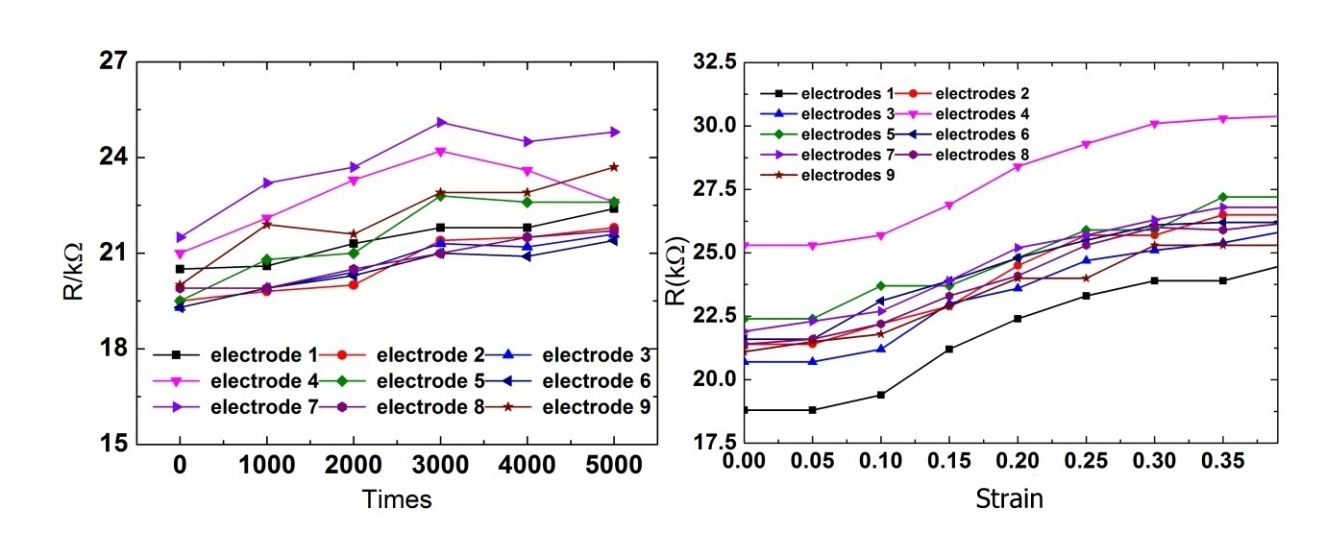


(a)

(b)

**FigureS2.**a, Resistance variation of 9 conductive lines upon repeated stretchto 200% for 5000 times. The resistances of all the lines increase steadily, though with some deviations. b,Resistances as a function of strain for those being pre-stretched by 100% for 1000 time, showing a linear relationship for a strain up to 40%.

1. **Resolution of the skin-like sensor**

The resolution is defined as the number of touch points that can be resolved per square inch (PPI). The skin-like sensor consisted of a crisscross conductive network. Each conductive line has a width of 10 µm with periodicity of 50 µm. Thus the potential pixel size of the fabricated skin-like sensor is 50×50 µm^2^, corresponding to a resolution of 1.79×10^5^ PPI. As each cross point of the skin-like sensor is equivalent to a receptor, the resolution of the skin-like sensor is about two orders of magnitude higher than the density (~1.56×10^3^ PPI) of touch receptors of human skin[^5^](#_ENREF_5). However the real resolution of the skin-like sensor measured is limited by the test method used. The skin-like sensor was attached to the electrodes that were connected to a peripheral circuitry. The line density of the electrodes is one line per millimeter (mm), allowing only one per twenty conductive lines used for the measurements and sensation tests, i.e. the resolution of the skin-like sensor tested is 625 PPI. But this is still higher than most reported skin-like sensor. Improvement of the testing resolution can be achieved by using microelectrode connects and will be the future work.

1. **Dynamic response**

The response of the resistance change of the skin-like sensor by a mechanical force was found very fast due to the nature of the electrical property of the conductive composite. Figure S3a shows the response of a conductive line subjected to a repeated mechanical force. Although the response to each deformation is rather noise mainly due to the poor electrode contact, it shows fast response and good repeatability. Figure S3b is a zoomed-in response of the measured voltage to a force, showing extremely fast response with the rising and falling times in the order of 25 ms, which is mostly limited by the measurement system.


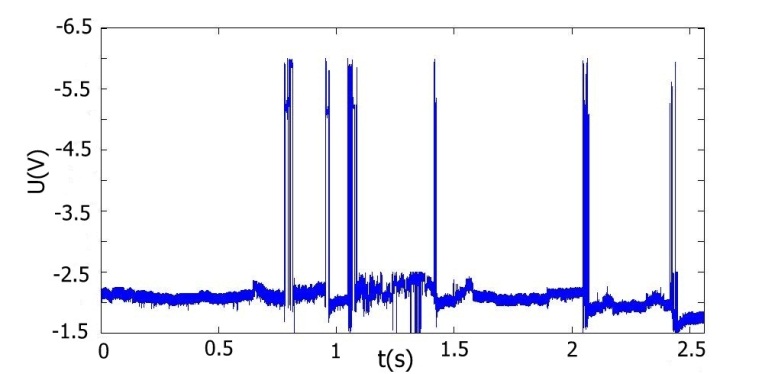


(a)


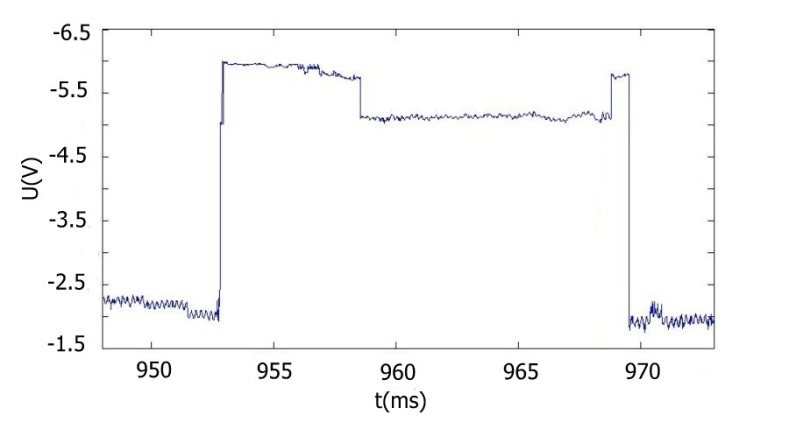


(b)

**Figure S3.** Response of a conductive line to a repeated pressing, showing the fast response in the order of milliseconds.

1. **TCR of the sensor**

TCR is defined as the relative change of the resistance, ΔR to the total resistance in a unit temperature range, ΔT, TCR=ΔR/(RΔT). Figure S4 shows the calculated resistance change as a function of temperature from Figure 3a.


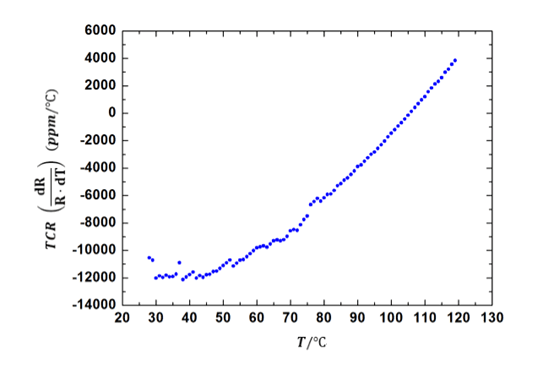


FigureS4. TCR of the skin-like sensor for the measurement of Figure 3

1. **Measurement circuit design**

The peripheral circuitry was composed of a microchip (MSP430F149), a 4-channel, 12-bits A/D converter (ADS7824), 4 decoders(CD4514B) and 4 analog multiplexers(CD4067B). The decoders were used to select the channels of the skin-like sensor and also provideda 5V voltage for resistance measurements. Each channel of the skin-like sensor was connected with a 10kΩ resistor in series, and the node voltage of the junction before and after the deformation of the skin-like sensor would pass through the multiplexer to the A/D converter. The result of the detection was read out from the microchip by serial communication through an USB cable to a PC.

**References**

1. Coutts, L., Bamber, J. & Miller, N. Multi-directional in vivo tensile skin stiffness measurement for the design of a reproducible tensile strain elastography protocol. *Skin.Res.Technol.***19**, 37-44 (2013).

2. Wang, Z. Polydimethylsiloxane Mechanical Properties Measured by Macroscopic Compression and Nanoindentation Techniques. (University of South Florida, 2011).

3. Agache, P.G., Monneur, C., Leveque, J.L. & Derigal, J. Mechanical-properties and young's modulus of human-skin invivo. *Arch. Dermatol. Res.***269**, 221-232 (1980).

4. E.R.P.Muthu, J. Mechanics of silicon micro needle penetration in human cadaver skin and skin substitutes. (Lehigh University, 2007).

5. Dahiya, R.S., Mittendorfer, P., Valle, M., Cheng, G. & Lumelsky, V.J. Directions Toward Effective Utilization of Tactile Skin: A Review. *IEEE Sensors J.***13**, 4121-4138 (2013).

6. Pan, L. et al. An ultra-sensitive resistive pressure sensor based on hollow-sphere microstructure induced elasticity in conducting polymer film. *Nature Commun.***5**, 3002 (2014).

7. Wang, C. et al. User-interactive electronic skin for instantaneous pressure visualization. *Nature Mater.***12**, 899-904 (2013).
